# A case of concomitant subclavian steal syndrome and thoracic outlet syndrome

**DOI:** 10.1016/j.jvscit.2024.101613

**Published:** 2024-08-22

**Authors:** Akihiro Kageyama, Taku Suzuki, Yasuhiro Kiyota, Noboru Matsumura, Takuji Iwamoto, Masaya Nakamura

**Affiliations:** Department of Orthopaedic Surgery, Keio University School of Medicine, Tokyo, Japan

**Keywords:** Endoscopic-assisted infraclavicular approach, Subclavian steal, Subclavian steal syndrome, Thoracic outlet, Thoracic outlet syndrome

## Abstract

A 59-year-old woman presented with dizziness and numbness of her left upper limb. Computed tomography (CT) angiography revealed an occlusion of the subclavian artery at its origin, leading to a diagnosis of subclavian steal syndrome. She was treated with percutaneous angioplasty and stenting; however, her symptoms did not improve. CT angiography of the arm in the elevated position revealed subclavian artery stenosis at the costoclavicular space, and the diagnosis was neurogenic thoracic outlet syndrome (TOS). First-rib resection was performed, and the symptoms disappeared immediately after surgery. TOS should be considered when symptoms persist despite subclavian steal syndrome treatment. Physical examination and CT imaging with the arm elevated aid in diagnosing TOS.

Subclavian steal syndrome (SSS) is characterized by stenosis or occlusion of the subclavian artery at the origin of the aortic arch and proximal to the origin of the vertebral artery. This condition results in reduced blood flow to the subclavian artery, leading to compensatory retrograde blood flow from the vertebral artery to the subclavian artery.^1^ Thoracic outlet syndrome (TOS) results from the entrapment of neurovascular structures in the thoracic outlet.^2^ Both conditions manifest with symptoms such as dizziness or numbness in the upper extremities.^2^, ^3^, ^4^, ^5^, ^6^, ^7^ Differentiating between these two diseases is challenging because of their similar symptoms. This report presents a rare case of concomitant SSS and TOS that was diagnosed only after treatment for SSS. The patient provided written informed consent for the publication of her case details and imaging findings.

## Case report

**Fig 1:**
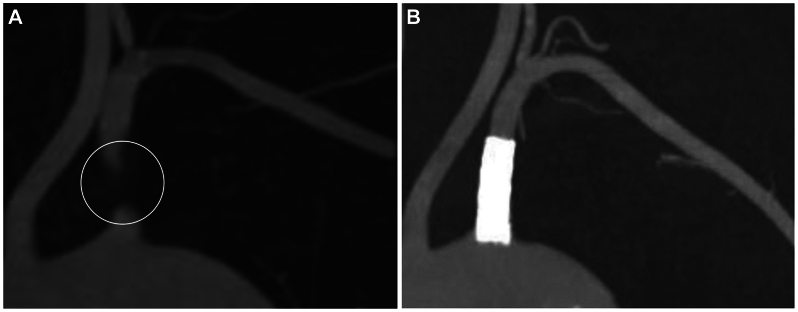
Computed tomography (CT) angiography with the arm dropped. Before **(A)** and after **(B)** the endovascular dilation with a stent.

A 59-year-old woman presented with dizziness and numbness of her left upper limb. The patient’s systolic blood pressure was 79 mmHg in the left arm and 108 mmHg in the right arm. She was diagnosed with hyperlipidemia and had a history of smoking 10 cigarettes per day for 38 years. Computed tomography (CT) angiography, with the arm dropped, revealed an occlusion of the left subclavian artery at the origin from the aortic arch and proximal to the origin of the vertebral artery (Fig 1, *A*), whereas ultrasound was not performed. The patient was diagnosed with SSS, likely due to atherosclerosis, by a cardiologist at another hospital. Subsequent endovascular dilation using a stent successfully expanded the origin of the subclavian artery (Fig 1, *B*). Although the blood pressure of the left arm improved to 108 mmHg post-procedure and that of the right side was 112 mmHg, the patient’s dizziness and numbness persisted. Despite thorough neurosurgical, neurological, otolaryngological, and ophthalmological examinations such as brain magnetic resonance imaging or positional nystagmus test, the tests were normal, and the cause of the dizziness remained unknown. The patient was then referred to our hand surgery hospital.

**Fig 2:**
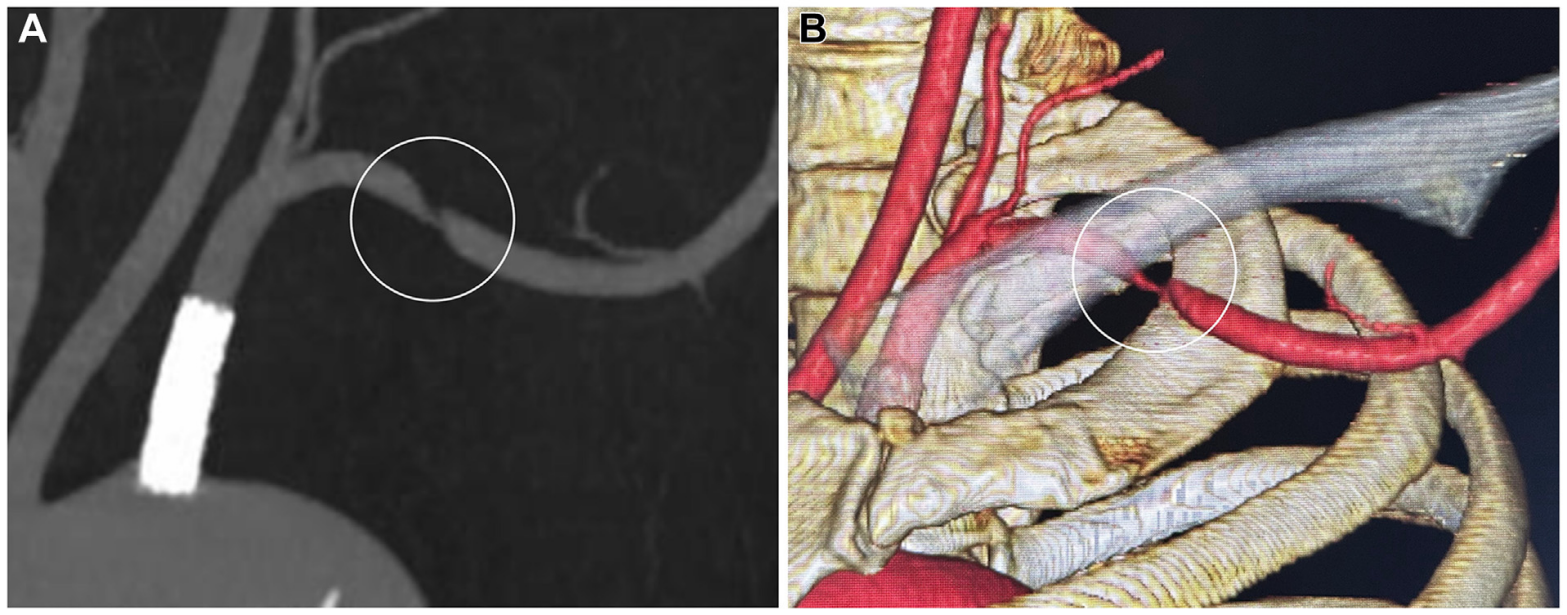
Computed tomography (CT) angiography with the arm elevated showing stenosis of the subclavian artery at the costoclavicular space.

The numbness worsened during the Roos test (90° abduction and external rotation of the shoulder). The grip strength on the left side was 12 kg compared with 25 kg on the right side. Tenderness and Tinel’s sign were observed in the supraclavicular fossa, which radiated to the upper arm, forearm, and hand, particularly in the ulnar distribution. Cervical spine radiography did not reveal any cervical or abnormal rib. CT angiography, with the arm elevated, revealed stenosis of the subclavian artery at the costoclavicular space (Fig 2, *A* and *B*). Based on clinical and radiographic findings, she was diagnosed with neurogenic TOS, and surgery was planned for endoscopic-assisted resection of the first rib and scalene muscles.^8^

**Fig 3:**
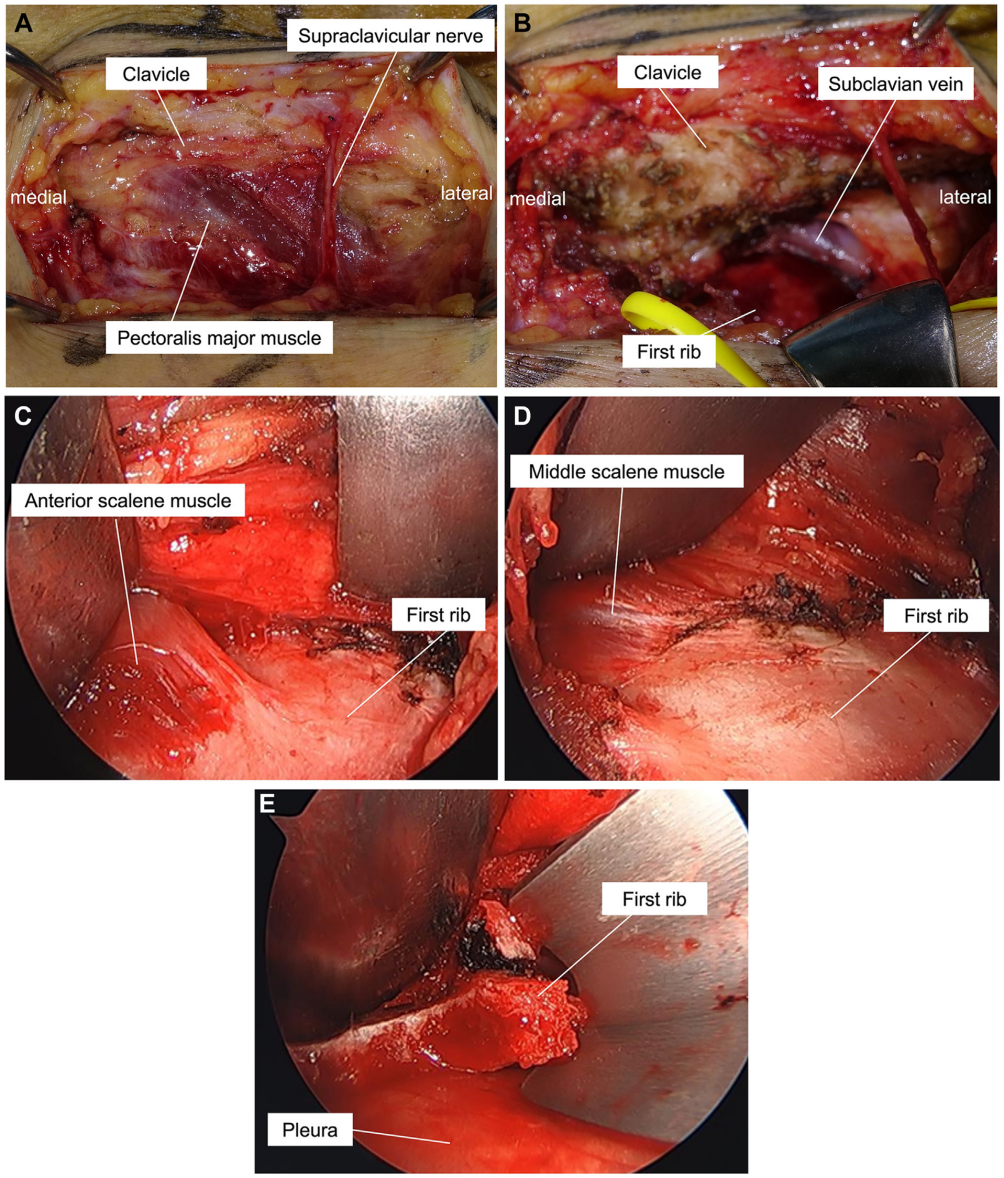
Operative findings. **A**, A transverse infraclavicular incision reveals the clavicle, pectoralis major, and supraclavicular nerve. **B**, The subclavian vein and first rib are revealed after the excision of the subclavian muscle. **C**, Endoscopic view of the first rib and anterior scalene muscle. **D**, Endoscopic view of the first rib and middle scalene muscle. **E**, Resection of the first rib.

A 7-cm transverse incision was made 1 cm below the clavicle, and the pectoralis major muscle was identified while preserving the supraclavicular nerves (Fig 3, *A*). The pectoralis major was released from the clavicle to expose the subclavian muscle, which was subsequently excised. Thereafter, the subclavian vein and first rib were exposed (Fig 3, *B*), and the subclavian vein was dissected to create a space between the vein and the rib. The subclavian vein, subclavian artery, and brachial plexus were protected using a muscle retractor.

**Fig 4:**
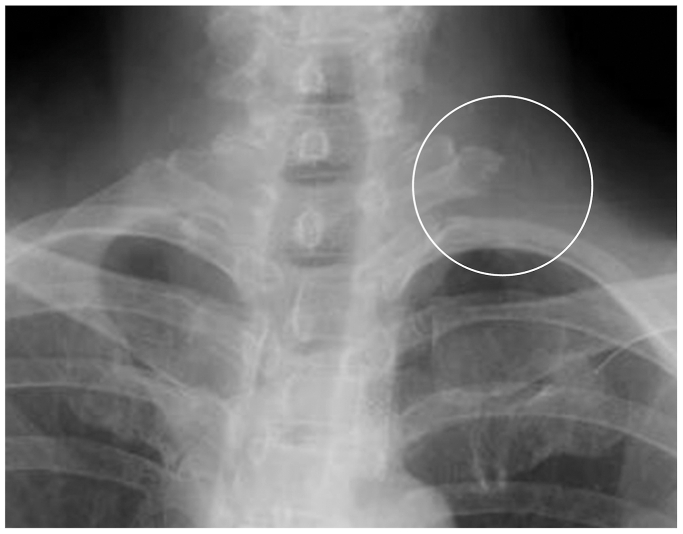
Postoperative radiographs of the first rib.

A 4-mm 30° oblique endoscope (1488 HD Camera System; Stryker) was inserted through the same incision, and the anterior and middle scalene muscles were released from their insertions at the first rib using electrocautery (Fig 3, *C* and *D*). Under direct vision, the anterior aspect of the first rib was elevated from the pleura and resected piece by piece using a rongeur to avoid damage to the pleura. Subsequently, the posterior aspect of the rib was elevated from the pleura and resected using a rongeur with the assistance of endoscopy (Fig 3, *E*). The first rib was resected near the transverse process of the T1 vertebra (Fig 4), and the pectoralis major was sutured to the clavicle.

**Fig 5:**
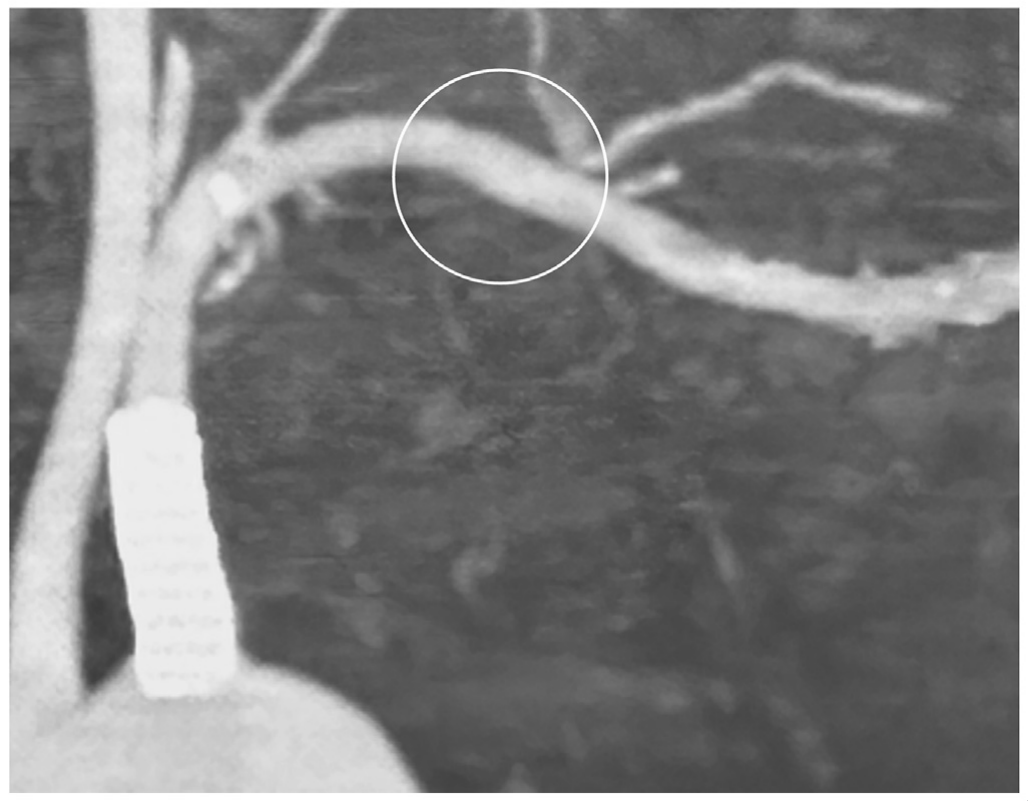
Postoperative computed tomography (CT) angiography with the arm elevated, showing an improvement of the subclavian artery stenosis at the costoclavicular space.

No postoperative complications, such as pneumothorax, hemothorax, neurovascular injury, or hematoma, occurred. Preoperative symptoms immediately improved a day after surgery. CT angiography with the arm elevated was performed 6 months postoperatively, and it showed an improvement of the subclavian artery stenosis (Fig 5). A follow-up examination at 12 months revealed no dizziness, numbness of the upper limb, or symptom recurrence. The patient’s grip strength improved from 12 to 23 kg on the left side. The Disabilities of the Arm, Shoulder, and Hand score improved from 90 points preoperatively to 4 points at 12 months postoperatively.

## Discussion

We reported a case of concomitant SSS and TOS that was not diagnosed until after treatment for SSS. Resection of the first rib and scalene muscles led to improvement of symptoms such as dizziness and numbness in the left upper limb.

As described above, SSS is characterized by a reversed flow in the vertebral artery in the context of proximal subclavian stenosis. Atherosclerosis is frequently the cause of this stenosis, and SSS predominantly affects men aged 50 to 60 years with a history of smoking.^6^^,^^7^ The retrograde flow of blood from the vertebral artery to the subclavian artery can lead to various symptoms. Cerebral ischemia may result in dizziness, diplopia, or dysarthria during limb movement, whereas limb ischemia can cause symptoms such as numbness.^3^^,^^6^^,^^7^

TOS and SSS are relatively rare syndromes, with reported prevalence ranging from 0.001% to 8% for TOS^2^^,^^5^^,^^9^ and 0.6% to 6.4% for SSS.^3^^,^^6^^,^^7^ To our knowledge, only two previous studies have reported the combination of SSS and TOS.^10^^,^^11^ In both cases, the individuals involved were women aged 34 and 40 years. One patient presented with TOS and SSS on the left side,^10^ whereas the other presented with TOS on the right side and SSS on the left side.^11^ Symptoms in both cases included dizziness and numbness of the upper limbs. Regarding the treatment of SSS, one patient underwent bypass grafting from the aorta to the subclavian artery,^10^ whereas the details of the other case are unclear.^11^ First-rib resection was performed for TOS in both cases.

TOS is commonly diagnosed in patients between the ages of 20 and 40 years, particularly for neurogenic symptoms.^2^^,^^4^^,^^5^ The symptoms of TOS are primarily characterized by numbness, pain, or fatigue in the upper limbs, resulting from compression of the brachial plexus or vascular bundle in the costoclavicular space.^2^^,^^4^^,^^5^ Compression of nerve bundles can also lead to symptoms related to autonomic nervous system dysfunction, such as dizziness or headaches, as observed in this case.^4^^,^^12^^,^^13^ Because the symptoms of TOS and SSS are similar, differentiating TOS before diagnosing SSS can be challenging. We believe that the dizziness was more likely due to compression of the brachial plexus in TOS. We believe that the treatment sequence should prioritize addressing the SSS first to improve blood flow at the base of the subclavian artery, as demonstrated in this case. It is crucial to consider TOS as a possibility when symptoms persist even after SSS treatment. In the diagnosis of SSS, CT is often performed with the patient’s upper extremity in dropped position. Physical examinations, such as the Roos test, or CT imaging showing subclavian stenosis with the arm elevated, can assist in the diagnosis of TOS. The infraclavicular approach provides excellent exposure of the subclavian vein, the anterior aspect of the first rib, and the anterior scalene muscle, which can cause compression of the subclavian vein.^14^ Accessing the posterior aspect of the first rib and middle scalene muscle is difficult without an endoscope due to the presence of the subclavian vein and narrow costoclavicular space. With endoscopic assistance, however, the posterior aspect of the first rib and middle scalene muscles can be visualized and resected.^8^ A disadvantage of the endoscopic infraclavicular approach is the difficulty of neurolysis owing to the narrow working space. This approach enables only resection of the first rib and scalene muscles.

## Conclusion

This report presents a rare case of concomitant SSS and TOS. It is crucial to consider TOS as a possibility when symptoms persist even after SSS treatment.

## Disclosures

None.
